# Supplementary material for: Optimal minimal residual disease threshold in pediatric acute myeloid leukemia: A retrospective cohort study based on the TARGET database
Source: PLoS Med. 2026 May 8;23(5):e1005088. doi: 10.1371/journal.pmed.1005088 (PMC13155632; doi:10.1371/journal.pmed.1005088)
Supplement: S1 Code — (ZIP) [file pmed.1005088.s002.zip › S2 code/PROJ8_8_tbl/PROJ8_8_tbl.htm]

## Cox Model

½á¾Ö±äÁ¿: First Event
Ê±¼ä±äÁ¿: EFS(years) 
 TIME.PHASE.4LMCOX : 1 
model:
Surv( X28 , X27 )~ X23.CS

|  |  |
| --- | --- |
| ¹Û²ìÊý£¨ÈËÊý£© | 1205 |
| ÊÂ¼þ·¢ÉúÊý | 592 |

±ÈÀýÔÖº¦Ä£ÐÍ

|  |  |  |  |  |  |  |  |  |
| --- | --- | --- | --- | --- | --- | --- | --- | --- |
|  | »Ø¹éÏµÊý(coef) | »Ø¹éÏµÊý±ê×¼Îó | z | Î£ÏÕ±È(HR=exp(coef)) | 1/Î£ÏÕ±È | 95%Çø¼äÏÂÏÞ | 95%Çø¼äÉÏÏÞ | P¡¡Öµ |
| X23.CS | 1.0024 | 0.0854 | 11.7336 | 2.7249 | 0.3670 | 2.3048 | 3.2216 | <0.0001 |

 rsq = 0.0968, maxrsq = 0.9987
 Log Likelihood £¨¶ÔÊýËÆÈ»Öµ£©= -3953.0660398348

|  |  |  |  |
| --- | --- | --- | --- |
|  | test | df | pvalue |
| Likelihood ratio test | 122.67477 | 1.00000 | 0.00000 |
| Wald test | 137.68000 | 1.00000 | 0.00000 |
| Score(log rank)test | 149.38098 | 1.00000 | 0.00000 |

±ÈÀýÔÖº¦Ä£ÐÍÕï¶Ï

|  |  |  |  |
| --- | --- | --- | --- |
|  | rho | chisq | p |
| X23.CS | -0.1607 | 14.7584 | 0.0001 |

Somers' Rank Correlation for Censored Data

|  |  |  |  |  |  |  |  |
| --- | --- | --- | --- | --- | --- | --- | --- |
|  | C | Dxy | aDxy | SD | Z | P | n |
| Model | 0.6055 | 0.2109 | 0.2109 | 0.0187 | 11.2653 | 0 | 1205 |

C: Harrell's C index; Dxy = 2(C - 0.5), Dxy is a U-statistic.
SD: Standard error of Dxy, so, SE. for C = SD/2 
 TIME.PHASE.4LMCOX : 2 
model:
Surv( X28 , X27 )~ X23.CS

|  |  |
| --- | --- |
| ¹Û²ìÊý£¨ÈËÊý£© | 537 |
| ÊÂ¼þ·¢ÉúÊý | 19 |

±ÈÀýÔÖº¦Ä£ÐÍ

|  |  |  |  |  |  |  |  |  |
| --- | --- | --- | --- | --- | --- | --- | --- | --- |
|  | »Ø¹éÏµÊý(coef) | »Ø¹éÏµÊý±ê×¼Îó | z | Î£ÏÕ±È(HR=exp(coef)) | 1/Î£ÏÕ±È | 95%Çø¼äÏÂÏÞ | 95%Çø¼äÉÏÏÞ | P¡¡Öµ |
| X23.CS | 1.5609 | 0.4650 | 3.3568 | 4.7630 | 0.2100 | 1.9146 | 11.8492 | 0.0008 |

 rsq = 0.0177, maxrsq = 0.3293
 Log Likelihood £¨¶ÔÊýËÆÈ»Öµ£©= -102.434322157296

|  |  |  |  |
| --- | --- | --- | --- |
|  | test | df | pvalue |
| Likelihood ratio test | 9.59577 | 1.00000 | 0.00195 |
| Wald test | 11.27000 | 1.00000 | 0.00079 |
| Score(log rank)test | 13.74276 | 1.00000 | 0.00021 |

±ÈÀýÔÖº¦Ä£ÐÍÕï¶Ï

|  |  |  |  |
| --- | --- | --- | --- |
|  | rho | chisq | p |
| X23.CS | -0.0151 | 0.0044 | 0.9471 |

Somers' Rank Correlation for Censored Data

|  |  |  |  |  |  |  |  |
| --- | --- | --- | --- | --- | --- | --- | --- |
|  | C | Dxy | aDxy | SD | Z | P | n |
| Model | 0.6544 | 0.3088 | 0.3088 | 0.1235 | 2.5001 | 0.0124 | 537 |

C: Harrell's C index; Dxy = 2(C - 0.5), Dxy is a U-statistic.
SD: Standard error of Dxy, so, SE. for C = SD/2 
Total:
model:
Surv( X28 , X27 )~ X23.CS +factor( TIME.PHASE.4LMCOX )

|  |  |
| --- | --- |
| ¹Û²ìÊý£¨ÈËÊý£© | 1742 |
| ÊÂ¼þ·¢ÉúÊý | 611 |

±ÈÀýÔÖº¦Ä£ÐÍ

|  |  |  |  |  |  |  |  |  |
| --- | --- | --- | --- | --- | --- | --- | --- | --- |
|  | »Ø¹éÏµÊý(coef) | »Ø¹éÏµÊý±ê×¼Îó | z | Î£ÏÕ±È(HR=exp(coef)) | 1/Î£ÏÕ±È | 95%Çø¼äÏÂÏÞ | 95%Çø¼äÉÏÏÞ | P¡¡Öµ |
| X23.CS | 1.0195 | 0.0840 | 12.1302 | 2.7719 | 0.3608 | 2.3509 | 3.2683 | <0.0001 |
| factor(TIME.PHASE.4LMCOX)2 | -19.7856 | 1059.5751 | -0.0187 | 0.0000 | inf. | 0.0000 | Inf | 0.9851 |

 rsq = 0.3311, maxrsq = 0.9936
 Log Likelihood £¨¶ÔÊýËÆÈ»Öµ£©= -4056.17187317927

|  |  |  |  |
| --- | --- | --- | --- |
|  | test | df | pvalue |
| Likelihood ratio test | 700.49058 | 2.00000 | 0.00000 |
| Wald test | 147.14000 | 2.00000 | 0.00000 |
| Score(log rank)test | 537.61739 | 2.00000 | 0.00000 |

±ÈÀýÔÖº¦Ä£ÐÍÕï¶Ï

|  |  |  |  |
| --- | --- | --- | --- |
|  | rho | chisq | p |
| X23.CS | -0.1347 | 10.7476 | 0.0010 |
| factor(TIME.PHASE.4LMCOX)2 | -0.5583 | 0.0000 | 0.9998 |
| GLOBAL |  | 10.7476 | 0.0046 |

Somers' Rank Correlation for Censored Data

|  |  |  |  |  |  |  |  |
| --- | --- | --- | --- | --- | --- | --- | --- |
|  | C | Dxy | aDxy | SD | Z | P | n |
| Model | 0.7525 | 0.505 | 0.505 | 0.015 | 33.6949 | 0 | 1742 |

C: Harrell's C index; Dxy = 2(C - 0.5), Dxy is a U-statistic.
SD: Standard error of Dxy, so, SE. for C = SD/2 
Created by EmpowerStats (www.empowerstats.com) and R on 2025-10-07
